# Researches on the Pathology and Treatment of Some of the Most Important Diseases of Women

**Published:** 1841-04

**Authors:** 


*Researches on the Pathology and Treatment of some of the most important Diseases of Women:* by Robert Lee, M. D., F. R. S., Physician-Accoucheur to the British Lying-in Hospital and the Saint Mary-le-Bone Infirmary; Lecturer on Midwifery in the School of Webb street; London, 1833. pp. 220.

The well-merited reputation of the author of this interesting practical work, together with the great importance of the subject treated of, must plead our apology for laying an analysis of it before our readers, so long after its issue from the press. In fact, though it has been before the profession eight years, we apprehend it is comparatively a stranger to our brethren of the West, on account of its not having been republished in this country, an honor, which it much more richly merits than some that have received it from our Atlantic publishers.

The diseases discussed by Dr. Lee, in the small volume before us, are such as very frequently befall the puerperal woman, viz.; Puerperal Fever, Phlegmasia Dolens, and Uterine Hemorrhage, which, whether we regard their fatality or the obscurity of their pathology, claim the most anxious attention of the accoucheur. Although, as we shall presently have occasion to remark with more particularity, the researches of our author have not irrefragably established the pathological views he advances, the cases and dissections, which he has given us, bestow upon his work its chief lustre and value. Not content with the observation of morbid manifestations during life, he has pursued disease, with praise-worthy zeal, into its most secret lurking places after death, and contributed materially to the elucidation of its seat and nature. Dispensing with a longer grace, we shall request our readers to sit down to the repast prepared for us. The work of Dr. Lee is divided into two parts; part first treats of Puerperal Fever and Crural Phlebitis; the latter appellation being used substitutively for *phlegmasia dolens*, which the author considers he has demonstrated to consist in inflammation of the veins of the thigh and leg; part second, of uterine hemorrhage. Chapter 1st. is taken up with introductory observations on the pathology of puerperal fever, the leading design of which is to show that in all cases of this disease, there is inflammation of the peritoneum or deep seated tissues of the uterus, its venous, absorbent, or muscular structure, and that the opinion, held by some very eminent men, that it is an idiopathic fever, *sui generis*, which produces inflammation in the uterine and other organs as its consequences, is to be rejected as untenable. This he has succeeded in doing very satisfactorily by a review of the histories of epidemic puerperal fevers, contained in the writings of such as have taken any adequate pains to investigate the ravages of the disease, by post mortem examinations. Puerperal fever must have existed from the most remote periods of antiquity; of this fact the records of medicine furnish abundant testimony; notices of it, however, are very cursory previous to its appearance as a malignant epidemic in the lying-in wards of the Hotel Dieu, about the middle of the seventeenth century. Since that period it has often occurred in the lying-in hospitals of Europe, in most of the large cities, and a great many sporadic cases have been observed. But, though the opportunities thus afforded have given rise to many *histories* of the disease, and the effects of remedies have been particularly noted, its pathology was but very imperfectly understood until very recently. Some, regarding it as a violent inflammation of the peritoneum, omentum, or other of the abdominal viscera, have recommended the most copious bloodletting and active purging for the treatment; others, referring all the symptoms, local and constitutional, to a specific, idiopathic fever, of a typhoid type, administer the most powerful stimulants and cordials. An inspection of the writings of such men as Hulme, Leake, Denman, Clark, Gordon, Hey, Armstrong, &c. will show this remarkable discrepancy of views and practice, and we should have remained in hopeless perplexity, destitute of such accurate knowledge as is necessary to furnish a clew to rational practice, if pathological anatomy had not been more carefully attended to, than it seems to have been by any of the distinguished writers, who have been mentioned. Dr. Lee had a clear perception of the chasm that remained, and of the necessity of farther researches to fill it. Alluding to these writers, he says:

"To reconcile their discordant statements, with respect to the nature and treatment of the affection, it appeared to me requisite, that it should be examined not only in hospitals, but also in private practice, for several successive years, throughout all the different seasons. In this manner did it seem only possible to ascertain, whether diseases had been described essentially distinct from one another, or merely varieties of the same affection, modified, perhaps, by some powerful, but unknown causes.

"From the 1st of January, 1827, to the 1st of October, 1832, one hundred and seventy-two cases of well-marked puerperal fever came under my immediate observation in private practice, and in the British Lying-in Hospital and other public institutions in the western districts of London. The symptoms and progress of these cases were watched with close attention; the effects of the different remedies employed were observed, and where death took place, I carefully examined the alterations of structure in the uterine and other organs.

"Of fifty-six cases which proved fatal, the bodies of forty-five were examined, and in all were found some morbid change, decidely the effect of inflammation, either in the peritoneal coat of the uterus, or uterine appendages,—in the veins,—or in the absorbents of the uterus, accounting in a most satisfactory manner for the constitutional disturbance observed during life. The peritoneum and uterine appendages were found inflamed in thirty-two cases; in twenty-four, there was phlebitis; in ten, there was inflammation and softening of the muscular tissue of the uterus; and in four, the absorbents were filled with pus. These observations are therefore subversive of the general opinion now prevalent, that there is a specific, essential, or idiopathic fever, which attacks puerperal women, and which may arise independent of any local affection in the uterine organs, and even prove fatal without leaving any perceptible change in the organization of their different textures. As the constitutional symptoms thus appear to derive their origin from a local cause, it would certainly be more philosophical, and more consistent with the principles of nosological arrangement, to banish entirely from medical nomenclature the terms, puerperal and child-bed fever, and to substitute that of uterine inflammation, or inflammation of the uterus and its appendages in puerperal women. Puerperal peritonitis and peritoneal fever, are terms not less objectionable than puerperal fever, for in many fatal cases there is no proof whatever of the existence of any morbid affection of the peritoneum."

The disclosures resulting from the anatomical researches of Dr. Lee are confirmatory of those of several late eminent French pathologists; Andral, Dance, Danyau, Tonnelle, Duplay, Dugès and Mde. Boivin all concur in representing that the inflammatory affection may attack separately or conjointly the peritoneal envelope, the proper tissue, or the veins and lymphatics of the uterus. This enables us to explain the discordant statements of previous observers, by some of whom we are assured that no vestige of inflammation exists in this disease, merely because the peritoneum was exempt from it; whereas, had their examination been extended to the deeper-seated structures, conclusive evidence of its existence would doubtless have been observed. The different constitutional effects, produced by the same pathological lesion, viz., inflammation, according as one or another of the uterine tissues may be its seat, will enable us, also, to reconcile the conflicting portraitures of the disease, and the opposite methods of treatment recommended by different writers. We postpone any particular remarks on this variety of constitutional affection, and the corresponding variety of treatment demanded, until we come to trace, with our author, the different forms of uterine inflammation, and in this place express our concurrence in the justness of his criticism upon the novel and peculiar views, emitted by Dr. Gooch, with regard to the nature of puerperal fever. Dr. Gooch contended that an affection of the peritoneum is an essential accompaniment of the disease, although he did not undertake to define what that affection is, because it is not uniform.

"The most remarkable circumstance," Dr. Gooch observes, "which the experience of the last few years has taught us about peritoneal fevers is, that they may occur in their most malignant and fatal form, and yet leave few or no vestiges in the peritoneum after death. The state of this membrane, indicated by pain and tenderness of the abdomen, with a rapid pulse, appears to be not one uniform state, but one which varies so much in different cases, that a scale might be formed of its several varieties; this scale would begin with little more than a nervous affection, often removable by soothing remedies, and, when terminating fatally, leaving no morbid appearances discoverable after death. Next above this, a state in which this nervous affection is combined with some congestion, indicated, in the cases which recover, by the relief afforded by leeches, and in the cases which die, by slight redness in parts of the peritoneum, and a slight effusion of serum, sometimes colorless, sometimes stained with blood. Above this might be placed, those cases in which there are in the peritoneum, the effusions of inflammation without its redness; namely, a pale peritoneum and no adhesions, lymph like a thin layer of soft custard, and a copious effusion of serum rendered turbid by soft lymph. Lastly, the vestiges of acute inflammation of the peritoneum, viz., redness of the membrane, adhesion of its contigous surfaces, a copious effusion of serum, and large masses of lymph."*

* An Account of some of the most Important Diseases peculiar to Women, by Robert Gooch, M. D., 1831.

There can be no doubt that women, in the puerperal state are liable to purely neuralgic attacks, accompanied with diffused pain of the abdomen, and a rapid soft pulse, which are best relieved by opiates and warm fomentations; and there is as little doubt that spasmodic contractions of the uterus, constituting after-pains, have been mistaken for puerperal fever. Such affections may, indeed, pass into inflammation, but until the transition takes place, there is no propriety in considering such attacks as puerperal fever, and they will yield most readily to anodynes. But the assumption is wholly gratuitous that the peritoneum is the seat of this nervous affection; it is much more probable that the proper tissue of the uterus is implicated. Dr. Gooch erred in restricting the seat of puerperal fever to the peritoneum; it may be perfectly intact, and yet the subjacent uterine tissues may exhibit the vestiges of the most intense and destructive inflammation.

"In investigating the morbid anatomy of this class of diseases, Dr. Gooch appears to have been satisfied with simply inspecting the peritoneal covering of the uterus; now I am strongly inclined to believe, that if he had carefully examined the uterine, spermatic, and hypogastric veins, the absorbents, the uterus, and its appendages, and the sub-peritoneal tissues, he would frequently have found acute inflammation or some of its consequences. With the phenomena of inflammation of the deep-seated structures of the uterine organs, he appears indeed to have been perfectly unacquainted, as they are not even alluded to in the course of his Essay, and are generally confounded with the effects of loss of blood. In a fatal case, examined by Mr. Stanley, it is indeed stated by Dr. Gooch, that no inflammation of the veins of the uterus was detected, but the symptoms had not been such during life as to render it probable that such a condition of the veins existed. The absence of increased vascularity of the peritoneum, and of lymph and serum in its sac, does not prove that the subjacent tissues are in a healthy state. That a nervous affection or congestion of the peritoneum should give rise to all the symptoms and consequences of fatal uterine inflammation, is not only highly improbable, but is wholly unsupported by facts. Had Dr. Gooch estimated more correctly the value of pathological anatomy, in investigating the nature of disease, and placed less reliance on the uncertain operation of remedies, he could not possibly have fallen into so many serious practical errors respecting puerperal fever, as well as some of the organic diseases of the uterine organs in the unimpregnated state."

All the observations which our author has made, according to his judgment, warrant the following general inference:

"That inflammation of the uterus and its appendages must be considered as essentially the cause of all the destructive febrile affections which follow parturition, and that the various forms they assume, inflammatory, congestive and typhoid, in a great measure depend on whether the serous, muscular, or venous tissue of the organ has become affected."

In chap. ii, Dr. Lee describes the changes produced by inflammation in the uterine organs subsequent to parturition, and points out the local and constitutional symptoms by which their morbid conditions are characterized during life and distinguished from some other affections to which they bear a resemblance. The following are the principal varieties of inflammation of the uterus and its appendages in puerperal women, noticed by our author:

"I. Inflammation of the peritoneal covering of the uterus and of the peritoneal sac.

"II. Inflammation of the uterine appendages: viz. the ovaria, fallopian tubes, and broad ligaments.

"III. Inflammation of the mucous and muscular, or proper tissue of the uterus.

"IV. Inflammation and suppuration of the absorbent vessels, and veins of the uterine organs."

It is very curious to observe the physiological independence, so to speak, of the different tissues, entering into the composition of the same organ; a vital modification in any one of them may be easily propagated until the whole of *it* is implicated, while another, with which it is in immediate proximity, and with which vascular connexion is held, escapes entirely, or manifests but little sympathy. Thus in the digestive organs, the mucous membrane may be phlogosed and even ulcerated, and the subjacent muscular and serous coats be entirely healthy; or all these tissues, in aggravated attacks, may be simultaneously involved. The same is true with reference to the pleura, pulmonary parenchyma, and mucous membrane of the organs of respiration; the brain and its membranes: and Dr. Lee's researches, corroborated by those of other pathologists, attest the same in relation to uterine inflammation:

"These varieties of uterine inflammation," says he, "may take place independently of each other, though they are most frequently met with in combination. Peritonitis seldom occurs without some inflammation of the uterine appendages; but I have found both these textures severely affected, while the muscular coat of the uterus, and the veins, were wholly exempt from disease. The venous and muscular tissues of the uterus, are also liable to attacks of severe inflammation without any corresponding affection of the peritoneal covering, though it most frequently happens that inflammation, when excited, either in the veins or muscular coat of the uterus, involves also the peritoneum."

The following remarks will be perused with interest, and enable us to understand the varying phases, and not less varying danger and fatality of puerperal fever, in different atmospherical constitutions: haply, they may clip the plumage of certain confident, boasting practitioners, who, because they have luckily encountered none but the least dangerous cases, proclaim their ability to vanquish the disease always, and wonder at the failures of their less fortunate, but equally competent brethren:

"Inflammation of the uterine organs, like inflammation of the lungs and other affections of a similar character which assume an epidemic form, occurs more frequently at one season than an other, and at one period the peritoneum is the tissue most commonly affected, while at other seasons, the deeper seated tissues are almost invariably found affected by inflammation. That there is no essential difference between these varieties of uterine inflammation is proved by the circumstance, that in the course of a few days, in the same ward of the British Lying-in Hospital, and in patients, who were placed in contiguous beds during the prevalence of the epidemic, when the disease appeared to be communicated from person to person, peritoneal inflammation, uterine phlebitis, and the other varieties enumerated, all occurred in their most characteristic form. In some patients the local and constitutional symptoms indicated the presence of acute inflammation of the serous covering of the uterus; and in those cases where active depletion was employed at the commencement of the attack, most frequently a speedy recovery took place. In other examples, at the onset of the disease, there was comparatively little pain in the region of the uterus, the pulse was from the beginning rapid and feeble and the symptoms were such as to contra-indicate the use of blood-letting and cathartics. Such cases usually terminated fatally in defiance of local bleeding and the exhibition of mercury and opium, and other remedies; and on examination after death, either the veins, the muscular structure, or the appendages of the uterus, were found to be the textures most frequently inflamed."

It becomes, therefore, a most interesting inquiry, and it behooves every practitioner of medicine to enter into it carefully, what are the signs or symptoms, characteristic of these different forms of uterine inflammation? And to these we are now to attend in company with our author: comtemplating them in the order in which they have been laid down. 1st. In inflammation of the peritoneal covering of the uterus, and of the peritoneal sac, there is great tenderness of the hypogastrium increased by pressure, withpyrexia, and these may be regarded as the pathognomonic signs of this variety of uterine inflammation. The inflammation begins in the peritoneal covering of the uterus, and before it spreads from thence, there is neither hardness, tumefaction, nor tenderness on pressue in any other portion of the abdominal cavity. It is only after the inflammation has spread to the peritoneal sac that the abdomen becomes painful, swollen and tympanitic; and it is then, also, that the digestive organs seriously suffer, and diarrhœa and vomiting of black or dark colored fluids follow, the pulse becomes extremely rapid and feeble, the tongue dry and brown, the lips and teeth covered with sordes. The following remarks of Dr. Lee, in regard to the development and symptoms of this variety of uterine inflammation, should be carefully remembered, particularly those at the close of the quotation;—for some, if we mistake not, are lulled into dangerous security, and flatter themselves that inflammation does not exist, because the lochia continue to flow and the breasts to secrete milk: or they suffer the disease to be fully formed, before suspicion is awakened because its onset is not announced by rigors and overwhelming pain:

"The manner in which the disease commences varies considerably in different individuals. The attack of pain is sometimes sudden, at other times the ordinary increased sensibility of the uterus, remaining after natural labor, passes insensibly into the acute pain increased by pressure, the chief pathognomonic symptom of this affection. Most frequently the accession of the disease is marked by rigors, partial or general, sometimes so slight as almost to escape notice, at other times so violent as to produce severe shivering of the whole body. The cold stage, after a longer or shorter duration, passes away, and is succeeded by heat of skin, suffusion of the countenance, acceleration of the pulse, and quick respiration, thirst, frequently nausea or vomiting, and vertigo or intense pain across the forehead. Cough is also a common symptom of the disease. The rigors precede, accompany, or follow the increased sensibility of the uterus. In some of the most severe cases there has been no distinct rigor; but a quick pulse, hot skin, and hurried respiration have rapidly succeeded to the uterine pain. In most of the fatal cases the countenance has, from the commencement, been anxious and pallid, and the extremities cold.

"There is no uniformity observable in the appearance of the tongue in puerperal peritonitis. It is sometimes entirely covered with a thin, moist, white, or cream-like film; at other times, it is of a deep red, or brown color in the centre, with a thick yellow or white fur on the edges.

"The lochia, are often entirely suppresed; in other cases, only diminished in quantity. In some instances, they have an offensive odor. The mammæ usually become flaccid; yet, in some fatal cases, the milk has been secreted until a short period before death. The urine is often pased with pain and difficulty."

It is of great practical importance, inasmuch as peritoneal inflammation requires to be timely met by efficient treatment, to distinguish it from intestinal irritation, after pains and hysteralgia, with which it may be confounded. The distinguishing symptoms of intestinal or gastric disorder have been very correctly pointed out by Dr. Lee:

"The pain is from the commencement of the attack diffused over the whole abdomen: it is rather a griping than acute pain, does not commence in the region of the uterus, and is but little, if at all, aggravated by pressure. The abdomen is generally soft, puffy, and distended. The tongue is loaded; there is thirst and headache; neither the lochia, nor the secretion of milk, are suppressed. The febrile attack is usually preceded by evident signs of derangement of the bowels, such as flatulence, nausea, vomiting, constipation, or diarrhœa. Puerperal peritonitis is developed in a large proportion of cases before the end of the fourth day after delivery, sometimes even within twenty-four hours; whereas this affection rarely appears until the termination of the first week."

It is not always so easy to distinguish between inflammation and hysteralgia or severe after-pains. The pain attending genuine peritoneal inflammation is liable to paroxysmal exacerbations, resembling in this respect after-pains, and these, if there is unusual sensibility of the uterus, may simulate inflammation. The correct rule of practice in such cases, which we have always pursued and are happy to find sanctioned by Dr. Lee, is, whenever there is doubt, to bleed and proceed otherwise as if the existence of inflammation were certainly ascertained. The well known pathological fact, already alluded to, that neuralgic may be transmuted into phlegmasial affection, in all the organs and tissues of the system, furnishes a sufficient warrant for the practice indicated, and besides, as Dr. Lee very justly remarks:

"There are few puerperal women, except those of a feeble and irritable constitution, or who have been previously exhausted by profuse hemorrhage, or some chronic disease, who are seriously injured by cautious depletion, local or general; and where death has followed the abstraction of sixteen or twenty ounces of blood from the arm, the fatal result may fairly be attributed to the disease, and to the neglect of the remedy rather than to its abuse. In cases of intestinal irritation, I have often found the local abstraction of blood followed by decided relief: and the same holds true with respect to the severe irregular pains without inflammation, which often occur subsequently to delivery, and do not yield to the ordinary means of treatment."

When the peritoneal covering of the uterus is inflamed, the uterine appendages, the ovaria, Fallopian tubes and broad ligaments, are very generally involved also; in only one case did Dr. Lee meet with an exception to this. But when the uterine portion of the peritoneum is only slightly affected, the uterine appendages may be more seriously diseased and even disorganized. Between the folds of the broad ligaments, Dr. Lee has observed effusions of serous or purulent fluids, as consequences of their inflamed conditions. The inflamed uterine appendages may become agglutinated together and, contracting adhesions with the peritoneum at the brim of the pelvis and the inflammation extending to the cellular membrane exterior to the peritoneum, occasion an extensive collection of pus, in the course of the psoas and iliacus internus muscles, similar to what takes place in lumbar abscess.

"In three other individuals under my care," says Dr. Lee, "who ultimately recovered, the purulent matter formed along the brim of the pelvis made its way under Poupart's ligament to the upper part of the thigh, and escaped through an opening formed in that region. In all of these cases, contraction of the thigh on the pelvis took place, which remained for several months."

Such terminations of inflammation constitute the *depots laiteux* of the French writers of the last century, and were very hypothetically attributed to the repulsion of the milk, or its absorption and deposition about the pelvic viscera. With regard to the diagnosis between inflammation of the uterine appendages and peritonitis, as they are generally more or less combined, it is not always possible to make it with certainty. Dr. Lee observes, however, that in the former,

"The pain is generally less acute than in peritonitis, and is principally seated in one or other of the iliac fossæ, extending from them to the loins, anus and thighs. On pressure, the morbid sensibility will be found to exist chiefly in the lateral parts of the hypogastrium. The constitutional symptoms at the commencement of the attack do not materially differ from those which mark the accession of peritonitis, being often accompanied with strong febrile action, which speedily subsides, and is suddenly followed by prostration of strength and other changes which characterize inflammation of the muscular and mucous tissues of the uterus."

With the view of illustrating the phenomena of puerperal peritonitis and inflammation of the uterine appendages, our author gives a number of interesting cases, with dissections, from which we select one or two, as instructive specimens.

"Mrs. Tiffin, æt. 32, No. 18, Mercer street, Long Acre. Delivered on the 7th of July, 1829. Labour natural. On the 9th, the uterus was felt above the brim of the pelvis large, and hard, and it was very painful on the slightest pressure; lochia and milk suppressed; pulse 110 and feeble; tongue white; bowels open. Slight relief followed the abstraction of fifteen ounces of blood from the arm, and the application of leeches to the hypogastrium. 10th July. The whole hypogastrium is now exquisitely painful, and the abdomen is swollen. Pulse more frequent. There has been much nausea and vomiting during the night. Bowels open. V. S. ad ℥xxiv. Eighteen leeches to the region of the uterus. 11th. Vomiting continues, abdomen less swollen, and pressure over the region of the uterus produces little uneasiness. Pulse rapid and feeble, respiration hurried, countenance sunk, occasional delirium. The whole surface of the body is now of a deep yellow color. She became gradually more feeble and died in the evening.

"*Dissection.*—Present, Drs. Sims, Clark, and Williams. The abdomen was distended by a great accumulation of air within the bowels; the peritoneal coat of the small intestines was red, and vascular: the peritoneum of the fundus, and anterior portion of the body of the uterus, was coated with albumen, and the sub-peritoneal tissue in this situation, contained a sero-purulent and gelatinous fluid. From the incisions made into the lower part of the body of uterus there escaped pure pus, but whether this flowed from the vessels, or muscular tissue, it was not easy to ascertain. Between the folds of the broad ligaments, there was a deposition of a gelatinous and purulent fluid, and both fallopian tubes were of a deep red color, softened, and their cavities filled with pus. The right ovarium was of the size of a common hen's egg, of a pulpy gelatinous consistence, and its healthy organization entirely destroyed. The whole presented the appearance of a soft, fibrous, vascular pulp; the left ovarium was similarly affected."

"Mrs. Gyde, æt. 22, Brewer street, Golden square, after a natural labour, was delivered of her first child, on the 26th June, 1830. She continued perfectly well till the 28th, when she was attacked with rigors, suppression of the lochia, and great tenderness in the region of the uterus. V. S. to ℥xii, and leeches to the hypogastrium were employed, and calomel and opium were administered internally, at short intervals, by Mr. Stocker of Welbeck street, who saw her on the evening of the attack. The symptoms were not, however, relieved by these remedies. The pain extended gradually over the whole abdomen, during the three following days. The pulse became extremely feeble and frequent. The countenance sunk, respiration hurried. Tongue covered with a brown fur. Constant retching and vomiting. Before death, which took place on the 7th of July, (the 11th day after her delivery,) the belly had become enormously distended and tympanitic.

"*Dissection.*—Three or four pints of dark colored sero-purulent fluid were contained in the abdomen. The peritoneal sac and great intestines were distended with a fœtid gaseous fluid. The uterus and its appendages, the omentum, and small intestines, were all imbedded in lymph, and their peritoneal coat exhibited the other signs of having been severely inflamed. Near the fundus uteri on the left side, immediately underneath the peritoneum, was a circumscribed deposit of pus, about the size of a nutmeg. Another abscess, of a similar description, was observed under the peritoneal coat of the uterus on the left side. The other tissues of the uterus were healthy."

It is to be remarked that the autopsy in these cases shows clearly that they were of a more than usually violent grade; in one, the inflammation extended to the sub peritoneal tissue which was infiltrated with sero-purulent fluid; in the other a small abscess was found in the same tissue.

The next variety of uterine inflammation, that involving the muscular tissue of the organ, is much more rapid and fatal than the one we have just noticed, and has been very generally overlooked by the English writers, though it has been accurately described by the French. In examining the uterine structures, with a view to detect the lesions it may have suffered, care is requisite not to mistake for morbid, appearances which are perfectly normal to the uterus, shortly after delivery The following description will convey a very good idea of the healthy appearances of the organ, under the circumstances noticed:

"For several days after delivery, where no disease of the uterus has supervened, its lining membrane is coated with a yellowish brown, dark red, or ash-grey colored layer of no great thickness, which seems to be formed chiefly of the fibrine of the blood with small portions of deciduous membrane; the os and cervix uteri are at this time of a deep red color, from blood extravasated under the lining membrane. Where the placenta had adhered, numerous dark colored coagula of blood are found to seal up the orifices of the uterine sinuses in the inner membrane, and frequently to extend a considerable distance into these veins. The clots of blood, one extremity of which hangs loose within the cavity of the uterus, are often connected with a large fibrinous coagulum, which entirely fills the fundus uteri, and every where firmly adheres to the innner surface of the organ. The dark-colored layer, which usually coats the inner surface of the uterus after delivery, has been supposed to be the result of gangrenous inflammation, and has been described as such by some pathologists."

But when the muscular tisssue has been the seat of inflammation, it is found, on removing the fibrinous layer, to be totally disorganized, of a dark purple, greyish or yellowish hue, and so softened in texture as to be torn by the gentlest efforts made in removing the parts from the body.

"In some cases," Dr. Lee informs us, "the inflammation has affected the greater part of the muscular structure of the organ; in others it has affected only the cervix of the uterus, or the part where the placenta had adhered, and the natural appearance of the muscular fibre has been lost. In other instances, depositions of pus have been observed, either immediately under the peritoneum, or between the fibres of the proper tissue of the uterus."

This affection has been described under the term putrescentia uteri and has been supposed by some German and French writers to be a peculiar, specific disease, distinct from and independent of inflammation. But Dr. Lee thinks it may be safely inferred that it is the product of the inflammatory process, from the usual effects of inflammation on the muscular tissue in other parts of the body, and from the frequent occurrence of this affection in combination with peritonitis, and other varieties of uterine inflammation. The symptoms which attend inflammation of the proper tissue of the uterus, as given by Dr. Lee, are, pain of the hypogastrium, diminution or suppression of the lochial discharges, and rigors with *rapid feeble pulse;* the countenance becomes pallid, with an expression of great anxiety and distress; severe headache and delirium may be present; the skin is hot and dry at first, but afterwards cold, and sometimes of a peculiar blue or sallow tinge; the respiration hurried, *with great prostration of strength:*

"The diagnosis of this variety of uterine inflammation," observes Dr. Lee, "particularly where it is complicated with peritonitis or phlebitis, which is frequently the case, is extremely difficult. The prostration of strength, and the alteration of the features, which often exist from the commencement, the feebleness and rapidity of the pulse, the irregular fœtid state of the lochia, are not such constant symptoms as to be considered pathognomonic, and they may arise from other causes. The most attentive consideration of the phenomena, will only lead to a probability as to the nature of the affection; and sometimes its existence cannot be determined during life. In all the cases of this affection which I have observed, the resources of nature and of art have proved equally unavailing in arresting its fatal course. The active inflammatory symptoms which have usually manifested themselves at the commencement of the attack, have passed speedily away, whatever plan of treatment has been adopted, and have been rapidly succeeded by symptoms of exhaustion. Where the disease has not been complicated with inflammation of the other tissues of the uterus, the symptoms have not been such as to indicate the necessity of venesection; and, in one case, where a considerable quantity of blood was abstracted from the system, death soon followed. In other cases, where an opposite plan of treatment was had recourse to, the fatal termination seemed to be less speedy, though equally certain."

Some cases related by Dr. Lee, as well as others referred to by him, render it probable that this disorganization or softening of the uterine tissue may take place during utero-gestation and give rise to spontaneous rupture of the uterus. A case of this description occurred likewise in this city, a few years ago, and was reported in the Louisville Journal of Medicine and Surgery, No. 1, for January, 1838, by Dr. Lewis Rogers, whose habits of pathological research afford a guarantee for the accuracy of his observations. We select but a single case from Dr. Lee, as a specimen of the symptoms and post mortem appearances, observed in inflammation of the proper tissue of the uterus:

"Mrs. Chapman æt. 36, No. 9, Belton street, Long Acre. Delivered on the 19th of August, 1830; labour easy. On the 24th, after drinking freely of porter, was suddenly attacked with a violent rigor, of long continuance, which was succeeded by acute uterine pain, headache, and great frequency of pulse. No remedies of any kind were employed until the 27th, when I was first called to see her. She had been delirious in the night. The pulse 130, soft and compressible. Hurried breathing, great prostration of strength. Tongue brown and furred; diarrhœa. Surface of the body of a deep sallow color. The hypogastrium was painful on pressure. The abdomen generally neither swollen nor tender.

"The symptoms became aggravated in the night and she died on the morning of the 28th."

Concerning the next variety, inflammation and suppuration of the absorbent vessels and veins of the uterus, Dr. Lee remarks:

"No pathologist in this country had observed a case of inflamed absorbents of the uterus before the month of July,

1829, when a fatal example of the disease occurred in St. George's Hospital. A woman æt. 30, in an advanced stage of pregnancy, was admitted into that hospital on July the 1st, under the care of Mr. Cæsar Hawkins, in consequence of sloughing of the skin covering a diseased bursa of the patella. The removal of the bursa was followed by great constitutional disturbance, and on the fourteenth, labour came on. Two days after, symptoms of uterine inflammation made their appearance, and on the eighteenth day death took place. Though the pain was relieved by bleeding, she never rallied after the attack. On examining the body, some puriform lymph was found in the pelvis, but there was no increase of vascularity in the peritoneum. In the broad ligaments some fluid was also effused, and on each side numerous large absorbent vessels were observed passing up with the spermatic vessels, to the receptacle of the chyle, which was unusually distended. All these vessels and the reservoir itself, were filled with pus; but that in the receptacle was mixed with lymph so as to be more solid; the vessels themselves were firmer and thicker than usual. The thoracic duct was quite healthy. The uterus was scarcely contracted and the internal surface of the lower half was soft and shreddy and in a state of slough. The upper part, where no pus was found externally, was also healthy, or nearly so on its inner surface."

Since the occurrence of this example, several cases have come under the observation of Dr. Lee, and a still greater number has been collected by the French pathologists. Inflammation of the absorbent vessels of the uterus is frequently combined with uterine phlebitis, and abscess in the proper tissue of the organ; and it must be borne in mind that pus may be contained in the absorbents without these vessels having been inflamed. During pregnancy the absorbents receive very great development and are endowed with great vital activity, to fit them for the performance of the office assigned them after parturition, viz.; the reduction of the organ to its normal unimpregnated dimensions. These vessels, therefore, must not be supposed to have suffered inflammation, unless evidenced by an alteration of their proper tunics—opacity, induration, softening, increased vascularity. The cases in which inflammation of the absorbents really exists appear to bear a very small proportion to those in which pus is found contained in them and the thoracic duct. In an article in the May number, 1836, of the *Archives Generales de Medicine*, M. Duplay informs us that during the year 1830 he had occasion to observe thirty-six cases of metro-peritonitis, with suppuration of the lymphatic vessels of the uterus, and he gives the following tabular view of the manner in which this alteration was combined with others. Among these thirty-six cases, along with the suppuration of the lymphatic vessels, there was 1st. The presence of sero-purulent effusion into the cavity of the peritoneum in 29 cases; 2d. Absence of the sero-purulent effusion, in 7 cases. Among the 29 patients who had sero-purulent effusion, the uterus and its appendages were sound in 3 cases; besides the sero-purulent effusion there were pseudo-membranous concretions and concrete pus on the inner face of the uterus in two cases; there were purulent infiltration of the sub-peritoneal tissue, of the meso-rectum, and mesentery; purulent collection in the ovaries, layers of pus on the internal surface of the uterus, softening with suppuration or gangrenous softening of the same surface, in 16 cases; besides these alterations, variously combined, pus was found in the cavity of the veins in 8 cases. Among the 7 patients, who offered no sero-purulent effusion into the peritoneal cavity, pus was found in the uterine veins, in the sub-peritoneal cellular tissue, or concrete pus on the inner surface of the uterus in 4 cases; absence of pus in the veins, but infiltration of the sub-peritoneal tissue and concrete pus on the internal face of the uterus in 2 cases; softening of the inner surface of the uterus without suppuration in one case. Thus it appears that in the 36 cases observed, in which pus was found in the absorbents, there was at the same time suppuration of the peritoneum, uterus, or its appendages in all the cases but one, and in this there was gangrenous softening of the internal surface of the uterus. M. Duplay thinks that a case given by Cruiveilhier (*Anat, pathol. du corps humain*, 13 *livr*, *p*. 5) is the only one furnished by the records of medicine, in which there was suppuration of the lymphatic vessels, with complete integrity of the uterus and peritoneum. We shall not pursue the inquiry, which is more curious than practical, but return to trace with our author the vestiges of inflammation in a different order of vessels, the uterine veins. Uterine phlebitis has now been so frequently observed, that we have become tolerably familiar with it, and though it is not always possible to verify it in its commencement, it gives rise afterwards to symptoms and consequences, which are peculiar to it:

"In women who have enjoyed good health," Dr. Lee observes, "during pregnancy, and in whom the process of parturition has been easily accomplished, uterine phlebitis occasionally commences within twenty-four hours after delivery, with pain more or less acute in the region of the uterus, accompanied or followed by a severe rigor, or a succession of rigors, suppression of the milk and lochial discharge, acceleration of the pulse, cephalalgia, or slight incoherence, with most distressing sensation of general uneasiness, and sometimes by nausea, vomiting, and diarrhœa. These symptoms, after a short duration, are succeeded by increased heat, tremors of the muscles of the face and extremities, rapid feeble pulse, anxious and hurried respiration, great thirst, with brown dry tongue, and frequent vomiting of green colored matters. The sensorial functions usually become much affected, and there is a state of drowsy insensibility, or violent delirium and agitation, which is soon followed by symptoms of extreme exhaustion. The whole surface of the body not unfrequently assumes a deep and peculiar sallow or yellow color, or a petechial or vesicular eruption appears on different parts of the body. The abdomen, also, sometimes becomes swollen and tympanitic, and some of the remote organs of the body, such as the lungs, heart, brain, liver and spleen, or the articulations and cellular membrane, and muscles of the extremities, suffer disorganization, from a rapid and destructive congestion and inflammation."

The suppuration and gangrene in remote organs or parts, whether produced by secondary inflammation or the transportation of purulent matters, are not less singular than characteristic consequences of uterine phlebitis. The nose may become black and gangrenous, the eyes become suddenly affected with destructive inflammation, destroying vision many days before death, and enormous deposits of pus may take place in the cellular membrane, in the neighborhood of the large joints, or within the capsular ligaments with erosion of the cartilages. M. Tonelle states that the integuments covering the deep abscesses resulting from uterine phlebitis, are always of a violet color, or present a peculiar characteristic tension and shining appearance. The pus is not bounded by walls as in common abscesses, but diffused by an insensible transition into the surrounding parts:

"All these affections," says Dr. Lee, "have a common origin, and cannot be referred to any other cause than to the morbid condition of the veins of the uterus. The purulent, or other secretions, formed by inflammation within the cavities of these vessels, probably produce the whole of the injurious effects now described, by entering the system and contaminating the mass of blood, in like manner as poisons do when absorbed into the body. It may be true, as some have supposed, though it cannot be demonstrated, that a certain number of the purulent particles fix themselves in the muscles and other parts, like globules of mercury injected into the veins, and that they become the focus, or centre, of an inflammation exactly circumscribed, which speedily runs on to suppuration."

Sometimes uterine phlebitis pursues a much more insiduous and obscure march, the local symptoms, pain or uneasiness in the uterine region, are overlooked or do not exist, and the disease is only manifested by the constitutional disturbance and destructive lesions in distant parts of the body, which it produces.

With regard to the veins which are most apt to be inflamed, the extension of the disease, and the morbid changes induced, Dr. Lee makes the following remarks:

"The veins which return the blood from the uterus, and its appendages, may be either wholly or in part inflamed; generally, however, the inflammation attacks the spermatic veins alone, and for the most part the one only on that side of the uterus to which the placenta has been attached; and it may either confine itself to a small portion of the vessel, or extend throughout its whole course, from the uterus to the vena cava. The usual consequences of inflammation of veins are then apparent, viz., injection and condensation of the cellular membrane in which they are embedded, thickening, induration, and contraction of their coats, and the deposition of lymph, mixed with pus and coagula of blood within their cavities.

"The same is the case with regard to the hypogastric veins, one only being generally affected. These veins are, however, rarely inflamed in comparison with the spermatic, and this would seem to depend on the latter veins being invariably connected with the placenta, to whatever part of the uterus it may happen to be attached."

Dr. Lee is of opinion that the inflammation of the veins, in uterine phlebitis, is traumatic and analagous to venous inflammation succeeding to amputations. This disease commences in the gaping orifices on the uterine surface, corresponding to the attachment of the placenta, by whose separation they are left open and a direct communication established between their cavities and the atmospheric air. Uterine phlebitis is most usually associated with inflammation of the other tissues of the uterus, and Dr. Lee says,

"Where the veins alone are inflamed, the peritoneal and muscular tissues remaining unaffected, there is often either no pain, or only a dull pain with a sense of weight in the region of the uterus, and no other local symptom by which the disease can be recognized. The uterus too may return to its usual reduced volume, or nearly so, and it is only on the accession of the constitutional symptoms, viz., rigors, prostration of strength, rapid feeble pulse, low wandering delirium, attacks of vomiting and diarrhœa, with brown parched tongue, and ultimately rapid and destructive inflammation of the eyes, and purulent deposits in the substance of the lungs, that the existence of this insidious and dangerous affection can be determined. If the substance of the uterus be affected, this organ remains above the brim of the pelvis, large, hard and painful on pressure, as in puerperal peritonitis."

We subjoin two cases of uterine phlebitis from our author—the first an example of the insidious and deceptive form of the disease; the second an exhibition of it as manifested in the average number of cases:

*Uterine Phlebitis, with ulceration of the Articular Cartilages, and purulent effusion within the Capsular Ligament of the right Knee Joint, &c.*—Mrs. Mayhew, æt. 33, was delivered in the British Lying-in Hospital, on the 2d of March, 1829, after an easy and natural labor. The placenta was expelled in a few minutes after the infant, and her situation seemed favorable until the third day after delivery, when a considerable discharge of blood from the uterus took place. From the 6th to the 20th of March, she made no complaint of uneasiness in any part of the body, though her strength rapily declined. The countenance was of a dusky yellow tinge; the heat of the surface slighly increased; the respiration was hurried, particularly on bodily exertion; and the pulse was above 130 and feeble; the tongue pale and glossy, with total loss of appetite, though at no period was there nausea and vomiting. Bowels open. The uterus gradually receded into the pelvis, and pressure over the hypogastrium produced no sensible uneasiness. The milk was secreted sparingly. The lochial discharge had a peculiarly offensive smell.

"From the 20th to the 28th, when she died, the prostration of strength increased, and the pulse became still more frequent and feeble. The respiration was extremly hurried, and she was incessantly harrassed with a hacking cough, and the expectoration of a frothy mucus. The abdomen continued soft and flaccid, and not affected by pressure. She, however, during this period, complained of excruciating pains in all the joints of the right superior extremity, and in the right knee joint, which was observed to be quite swollen, but not discolored. This patient quitted the Hospital on the 23d, and was under the care of Mr. Armstrong, of Golden square, from that time until the 28th. Dr. H. Davies and Mr. Armstrong were present when I examined the body.

"*Appearances on Dissection.*—On laying open the abdomen, the intestines and other viscera presented a perfectly healthy appearance, and the uterus was found reduced to its ordinary size a month after delivery. On careful examination of the peritoneal coat of the uterus, a slight adhesion was observed between it, and the rectum on the left side. The uterus being removed, and its cavity laid open, a portion of what appeared to be placenta, about the size of a large nutmeg, in a putrid state, was seen adhering to its inner surface, at the part corresponding with the adhesion between the peritoneal coat and rectum. The muscular tissue of the uterus around this was of a dark color, approaching to black, and as soft as sponge. On cutting into it, about a teaspoonful of purulent matter escaped from the veins, and a small additional quantity was forced out from them by pressure. Small coagula of blood and lymph, plugged up the surrounding veins. The spermatic, and other abdominal veins, presented no morbid appearance, and the uterine appendages were healthy.

"On opening the capsular ligament of the right knee joint, where a fluctuation was perceived, about six ounces of thin purulent fluid escaped, and the cartilages of the joint were observed to be softened and extensively eroded. There was no appearance, however, of inflammation exterior to the capsular ligament, and the femoral vein was healthy.

"The right wrist was swollen, but the structure of the joint was not affected. The cellular membrane around it was unusually vascular and infiltrated with serum.

"Mrs. Keene, æt. 31, No. 6, Draper's place, Euston square, after a protracted labour of three days, was delivered on the 14th of July, 1829, by artificial aid, of a still-born hydrocephalic child. Immediately after the expulsion of the child, she was seized with a fit of the most intense shivering, which continued upwards of an hour, notwithstanding the exhibition of the most powerful stimuli; and the exhaustion which followed was so alarming that her life was despaired of. She rallied, however, and passed a quiet night. On the following, and two or three subsequent days, the shivering fits returned at irregular periods, sometimes in a slight form, at others, in that of a severe rigor, followed by a flush of heat, and partial or general perspiration. During this time, the effects consequent to parturition proceeded as usual. The uterus slightly painful on pressure; lochia natural; bowels open; pulse from 133 to 140, and extremely feeble. No complaint of uneasiness, with the exception of a troublesome cough and hoarseness, with which she has been afflicted during the latter months of pregnancy. On the 4th day from delivery, the secretion of milk appeared, for a short period, and afterwards receded. From this day to the 10th, the following were the symptoms: pulse rapid; skin universally of a dusky yellow color, and the heat of the surface increased; respiration hurried; thirst; tongue dry, but not furred; great prostration of strength; sallow and haggard countenance; restless and sleepless nights; mental faculties undisturbed. The uterus had gradually subsided, and no pressure, however great, either on it, or on the parts in its vicinity, caused pain, except in the right iliac region, where some uneasiness was felt; the flow of lochia natural; bowels regular. At this period, the hacking cough which had so long troubled her, became more frequent, and it was with difficulty she expectorated the ropy mucus which followed it, and which in the day amounted to an ounce. From the 11th day the respiration became more short and hurried; the pulse more rapid; occasional flushes of heat; thirst; extreme debility; diarrhœa. Pressure over the whole abdomen gave no uneasiness, nor was pain felt in any part of the chest, though auscultation plainly indicated the existence of disease, particularly on the right side. The patient made no complaint but of weakness, and of the cough. On the 12th, the dispnœa increased, and she sunk exhausted in the evening.

"*Dissection.*—Mr. Prout, surgeon to the British Lying-in Hospital, who had carefully observed the progress of the symptoms, from the period of delivery, was present with me when I opened the body. The uterus was of the size it usually is, about the second week after delivery, and exhibited externally no vestige of disease. On laying it open, its internal surface, as well as its muscular tissue, appeared also healthy, and the veins being traced, the right spermatic alone was found greatly enlarged and indurated. The uterus being removed from the body, for more minute examination, an incision was made into the right superior angle, to which the placenta had been attached, and here its veins were discovered to be empty, and their internal surface of a scarlet color. On tracing them towards the trunks of the right spermatic vein, they were found to contain sanious purulent fluid, and were contracted in their diameters, and coated with false membranes. The veins of the right ovarium and fallopian tubes were all plugged up with firm coagula. The spermatic itself was lined throughout its whole extent with dense membranes of a reddish or of an ash-grey color. Its coats, independent of these membranes, were of extraordinary thickness and firmness; and more like those of a large artery than of a vein. Its whole cavity was contracted; in some parts occupied by a dark colored fluid, in others, quite obliterated by adhesions, formed between the surfaces of the membranous layers deposited within it. At the termination of the spermatic in the vena cava, its orifice was scarcely large enough to admit a crow quill; traces of inflammation extended beyond this orifice, the vena cava being partially lined from two or three inches above it, with an adventitious membrane, strongly adherent to its coats, which were at this part double their natural thickness. In its passage upwards, the inflammation had extended a short distance into the right emulgent vein, which near its orifice was coated with a pellicle of lymph. On opening the thorax, a stream of air escaped from the right side; the lungs were collapsed, and upwards of two pints and a half of a red colored serum were found in the sac of the pleura. The right inferior lobe was coated with lymph, and a portion of the pleura on the anterior surface was destroyed, and a black gangrenous slough exposed in the substance of the lung. The pulmonary texture around was condensed, and of a deep violet or livid color. The left inferior lobe was also partially coated with a thin layer of lymph, and the pleura at one point on the anterior surface was elevated, as if by a small hard globular body beneath it. When this was laid open, it appeared to consist of a thick yellowish colored cyst or capsule, containing a soft black matter like a gangrenous eschar. The substance of the lungs around was unusually dense, and of a dark livid color."

In retrospecting calmly and attentively the different varieties of uterine inflammation, which have now been placed with sufficient distinctness before our readers, the first reflection suggested is, that we have no diagnostic signs by which it can be ascertained, with any degree of certainty, whether the muscular, nervous or absorbent tissue may be chiefly affected. Inflammation of the deep-seated textures may be so variously combined, and the symptoms to which they give rise are so entwined and confounded, that it is impossible to determine whether one is exclusively affected, or which is principally implicated. But it must be very apparent to the reader, though it may not have arrested his attention, that there is a striking difference in the local symptoms and constitutional affection, between the inflammation of these deep-seated structures and that of the peritoneal envelope of the uterus. In the former there is not from the first any thing like open reaction, or sthenic excitement; but the symptoms are of the typhoid kind *ab origine;* the pulse is *feeble* as well as rapid from the commencement, there are muscular tremors and profound prostration. In the latter, the ordinary signs and consequences of inflammatory action are exhibited; there is acute pain and tumefaction of the uterus, tenderness on pressure, the pulse though frequent is fuller, stronger, and more resisting; there is less disturbance of the sensorial or nervous functions; in a word, the febrile disturbance of the system is synochal, and the lancet and other depletory remedies are well borne, and must be used with freedom. Therapeutically considered, the former is widely different; neither the local nor constitutional symptoms indicate the abstraction of blood, nor can depletion be practised but at the imminent risk of irrecoverably prostrating the system. This distinction, which it is practically important to draw, is made by Dugès and Mde. Boivin, (*Traite Pratique des maladies de l'uterus, &c. Tome ii, p.* 231.) who describe an inflammatory and typhoid form of puerperal fever. It would be better, perhaps, to denominate them sthenic and asthenic, because doubtless in both there is inflammation, but its manifestations and effects are modified by the differences between the tissues that are the seat of the morbid affection.

Passing over *Chap. iii*, which treats of the *causes of uterine inflammation in puerperal women*, because it contains nothing but what is familiar to the profession, we take up *Chap. iv*, in which the *treatment* is discussed with perspicuity as well as commendable brevity.

The treatment of puerperal inflammation of the uterus may be divided into first, that which is applicable when the peritoneal coat is affected; and second, that which is required when the deep-seated tissues are invaded, or, if it be preferred to retain the appellation, puerperal fever, the treatment of the sthenic and asthenic varieties of the malady. In the first variety, practitioners are very generally agreed as to the indispensable necessity of bloodletting, both general and local, which, to be efficient, must be early and vigorously employed. The remarks of Dr. Lee on this topic are so excellent that we cannot refrain from quoting them:

"When the symptoms of puerperal peritonitis manifest themselves as before described and in a violent form, twenty or twenty-four ounces of blood should be immediately abstracted from the arm by a large orifice, and while the patient has the trunk and shoulders considerably elevated in bed. We should not be deterred from employing the lancet because the pulse is small and contracted, provided it does not exceed 110 or 115 pulsations in the minute: for in many cases the pulse has become fuller and stronger during the time the blood has been flowing, or soon after, and there has been a marked relief from suffering, In all cases, if possible a decided impression should be made upon the system, and where syncope or fainting follows the venesection, it increases the salutary effect. In no case of inflammation of the peritoneal surface of the uterus have I observed any bad consequence to result from depletion carried to this extent, and in many from its early use, the force of the disease has at once been completely broken."

In violent attacks, or where the pain is but slightly relieved by the vensection, Dr. Lee directs the application of one, two, or three dozen leeches to the hypogastrium, proportioning their number to the urgency of the symptoms,—the bleeding to be promoted, after the leeches have come off, by fomentations, or by a thin warm linseed meal poultice. There are few cases, in his opinion, in which it is necessary to have recourse to a second bleeding from the arm, and when the propriety of this is indicated by the recurrence of acute pain, the quantity of blood should not exceed ℥xii or ℥xiv. On this subject it is proper to observe that the greater number of western physicians have it not in their power to employ leeches in their practice, and therefore, under the circumstances specified by Dr. Lee, they are compelled to repeat the general bloodletting, and they should use the remedy a second time with more boldness than he allows. If a repetition of the remedy be clearly called for by the symptoms, it should be used for *effect* and not under the restraint imposed by arbitrary prescription as to *quantity*. Unless this second bleeding be carried to the extent of again making a decided impression upon the circulatory system, so as to allow the engorged capillaries to relieve themselves by contraction, it had better not be practised at all, as it would only serve to exhaust the patient. Let it not be inferred from these remarks that we are laboring under that degree of *sanguimania*, which prompts to the rash unsheathing of the lancet, or its employment in cases of doubtful propriety. With Dr. Lee we entirely agree that seldom, if ever, is it necessary or safe to bleed a third time, and in a very large proportion of cases a single bleeding will suffice.

Immediately after the bleeding, Dr. Lee directs eight or ten grains of calomel, in combination with five grains of antimonial powder and grs. 1½ or grs. ii of opium, or with ten grains of Dover's powder, and this should be repeated every three or four hours, until the symptoms begin to subside. In many of Dr. Lee's cases upwards of fifty grains of calomel have been given with decided benefit, and in two only out of one hundred and seventy cases has the mouth been severely affected. When the calomel and opium have subdued or mitigated the pain, a purgative enema or cathartic draught should be administered to open the bowels freely, when in some cases the uterine pain completely subsides.

*Blisters* to the hypogastrium and inside of the thighs and legs, Dr. Lee has often found advantageous, where pain in the uterine region has continued severe, even after general and local bleeding, and our own experience is in their favor. We have, also, obtained highly salutary effects from the external use of spts. turpentine over the hypogastric region.

On the recommendation of Recolin, Dance, and Tonellé, Dr. Lee has on several occasions tried injections of warm water into the vagina and cavity of the uterus, and, as he thinks, with decided advantage. These injections, repeated three or four times in the course of the day, they affirm, not only wash away the putrid matters adhering to the internal surface of the organ, but appear to relieve the irritation and inflammation of the organ itself.

To relieve the severe and distressing irritation of the stomach that not unfrequently comes on in the progress of uterine inflammation, our author has found ten grains of the sub-carbonate of potash in an ounce of aqua menth. virid., given every two or three hours, much more effectual than anodynes and effervescing draughts. Should diarrhœa take place, it is best controlled by opium in the form of the starch and laudanum glyster.

Such is a sketch of the treatment of puerperal peritonitis, recommended by Dr. Lee, and we can aver that it is as efficacious as it is simple. It is substantially the practice which we have pursued for many years, and with as much success, to say the least, as any other method of treatment can claim. We are fully aware that the *purgative* branch of the treatment has been pursued in a very different manner by many practitioners with, as they report, very favorable results: calomel, salts, sena, tartarized antimony, &c. being exhibited so as to produce and continue very active catharsis. But it has always appeared to us that such irritating purgation is decidedly pernicious, and that opium, so much reprobated by some, can hardly be dispensed with in the treatment of this painful malady.

During the first or inflammatory stage of the disease, tonics and stimulants are inadmissible; but when symptoms of exhaustion supervene, wine, ammonia, camphor, quinine, &c. may be resorted to and sometimes with the happiest effects. As we believe that physicians are too generally disposed to abandon their patients in despair, when such symptoms set in, the encourgement to perseverance, which Dr. Lee holds out, should not be forgotten:

"I cannot too strongly urge the necessity of continuing to employ these remedies whilst the slightest hope of recovery is entertained. I have seen several patients restored to health, where the pulse had risen to 160, and was so feeble as scarcely to be felt at the wrist, where there was constant delirium, and the most alarming prostration of strength. Recovery has even taken place, in some cases which I have observed, where the abdomen has become tympanitic, and effusion to a considerable extent taken place in the abdominal cavity. In no acute disease is it of greater consequence than in this now under consideration, that the patient should be visited by the medical attendant at short intervals, and that the effects of the remedies he prescribes should be narrowly watched."

With regard to the treatment of inflammation of the deeper-seated tissues of the uterus, viz. the absorbents, veins, or muscular structure, the symptoms from the commencement, as has already been intimated, are such as to contra-indicate the use of bloodletting. According to Dr. Lee's experience, even when the reaction has been violent, the relief obtained by venesection has only been temporary, and in some instances the abstraction of a small quantity of blood from the arm has produced alarming syncope, nor do his observations warrant the conclusion that we are in possession of any remedial means on which much reliance can be placed. The French physicians, however, have great confidence in mercury, employed so as to excite salivation. On this practice Dr. Lee thus comments:

"In several cases of uterine phlebitis, I have employed this remedy to a great extent, externally, and speedily brought the system under its influence; yet the progress of the symptoms was not arrested, and the patients died as others had done where the mercury had not been administered. In other cases I have employed mercury to a great extent, internally, without the slightest benefit; and it may justly be doubted, from the results of M. Desormeaux's practice, whether or not it possesses the influence M. Tonellé supposes, for of forty-three cases where mercury was used by him as the chief remedy, only fourteen recovered. In the latter stages of inflammation of the deeper-seated structures of the uterus, the great depression of the powers of the system renders the liberal administration of stimulants absolutely necessary, and in several cases of phlebitis the life of the patient appeared to be preserved by them."

Our author closes his observations on puerperal fever with some very judicious reflections on the prophylactic treatment, which is entitled to the closest attention at all times, seeing the formidable and too often uncontrollable character of the disease when once excited. His directions are founded upon the proper basis for puerperal hygiene, viz. the recognition of the principle that a woman recently delivered is in a state of predisposition to inflammation of the uterus and its appendages. This will itself suggest the proper cautionary precepts, and instead of even enumerating these we prefer to dilate a little on a single one of them but slightly noticed by Dr. Lee, viz. the risk incurred by the administration of active or acrid cathartics soon after delivery. The tendency of such practice is always to awaken any latent predisposition to irritation in the uterine organs, and in many instances to excite inflammation. None but the mildest aperients should be prescribed; even these should be deferred as long after delivery as possible, and better still will it be by evacuating the bowels in the onset of labor or just before it sets in, to dispense with them altogether. We fear that on this important subject, correct views are not as generally disseminated and acted on as they ought to be, and that there is a deterioration of medical practice since the good old days of Smellie,—his "breathing sweats" and "warm caudle" being infinitely preferable to senna, scammony, aloes and colocynth.

H. M.

(*To be continued.*)

